# *p*‐Values Are Not Evidence: Navigating the Gap Across Modes of Inference

**DOI:** 10.1002/ece3.74419

**Published:** 2026-10-25

**Authors:** Hallie E. Arno, Robert J. Blackmore, Samantha E. Crowley, S. M. R. Evon, Ian A. Fleming, C. Tyler Fountain, Stephan Hamisch, Emily M. Monk, Alec L. Robitaille, David C. Schneider

**Affiliations:** ^1^ Department of Ocean Sciences Memorial University of Newfoundland and Labrador St. John's Newfoundland and Labrador Canada; ^2^ Research Initiatives and Services Memorial University of Newfoundland and Labrador St. John's Newfoundland and Labrador Canada; ^3^ Department of Biology Memorial University of Newfoundland and Labrador St. John's Newfoundland and Labrador Canada; ^4^ Department of Geography Memorial University of Newfoundland and Labrador St. John's Newfoundland and Labrador Canada; ^5^ HGF‐MPG Joint Research Group for Deep‐Sea Ecology and Technology Max Planck Institute for Marine Microbiology Bremen Germany; ^6^ HGF‐MPG Joint Research Group for Deep‐Sea Ecology and Technology, Alfred Wegener Institute, Helmholtz Centre for Polar and Marine Research Bremerhaven Germany

**Keywords:** likelihood, likelihood ratio, *p*‐value, statistical inference, statistical significance

## Abstract

The use of *p*‐values and statistical significance testing as the primary mode of inference in science has been long debated among statisticians and more recently among ecologists. Ecological literature has discussed embracing other modes of inference, such as Bayesian statistics and the use of direct likelihood inference. We reviewed ecology, evolution, and environmental science literature covering the past three decades to investigate trends in the use of different modes of inference over time and to assess the degree of discrepancy between reported *p*‐values and calculated likelihood ratios. *p*‐Values and likelihood ratios were found to scale in a more direct way for single degree of freedom tests, but in a more complex manner for models with more than one parameter. These results demonstrate the degree to which the likelihood ratio (a measure of evidence) differs from a *p*‐value, a measure of uncertainty that is widely presented as a measure of evidence among publications within the fields of ecology and evolution. Additionally, our investigation revealed that modes of inference within ecological studies changed little over the 20‐year time period, indicating a failure to adopt alternative modes of inference. We also found downward trends in the reporting of *p*‐value thresholds (e.g., “*p* < 0.05”) in two journals, with no change in three others. The prevalence of the divergence in the measure of evidence and the *p*‐value measure of uncertainty remains to be confirmed in other journals, time points, and subfields. An overreliance on *p*‐values alone can alter the conclusions drawn from research, impacting management of species and ecosystems. We recommend that authors report both a measure of evidence and uncertainty, and justify their mode of inference in an attempt to produce statistical results that are comprehensive, replicable, and reliably interpretable.

## Graphical abstract

Likelihood ratios have been suggested as an alternative to *p*‐values in ecology and environmental science, but the differences between these two statistics has not been thoroughly examined. We searched environmental literature and found that *p*‐values and likelihood ratios do not necessarily scale linearly, and suggest authors report both.

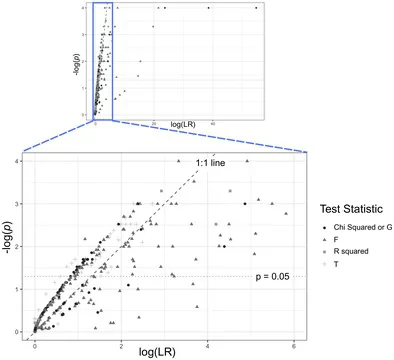

## 1 Introduction

Ecologists and evolutionary biologists have traditionally relied on frequentist methods, such as the *p*‐value, to draw conclusions from data. *p*‐Values, which measure the probability of an observation as or more extreme if the null hypothesis were true, are not necessarily the best‐suited method for many types of ecological data (Greenland et al. 2016; Halsey 2019; Hobbs and Hilborn 2006). For example, many ecological studies are observational rather than experimental, where the statistical null hypotheses do not reflect the most biologically relevant or meaningful question (Anderson et al. 2000). Additionally, ecological studies often have non‐independent observations, repeated measurements, and many confounding factors, which can artificially lower *p*‐values, making them inappropriate and uninformative (e.g., Davies and Gray 2015; Peacor et al. 2025). *p*‐Values control for Type I error (false positives). In life and environmental sciences, it may be equally important to control for Type II error (false negatives)—in many ecological restoration and conservation projects, the cost of inaction is high (Mapstone 1995). For example, a researcher might conclude that a non‐significant *p*‐value fails to reject the null hypothesis and therefore there are no differences between two populations of endangered species, while the addition of another statistic, such as a likelihood, might more transparently reveal slight differences, particularly in a small sample (Brosi and Biber 2009). The statistical methods used to determine which results are “significant” can impact management actions (Walters and Hilborn 1978), and debate around decisions on the significance of results has a long history in ecology (Burnham and Anderson 2014; Murtaugh 2014; Stanton‐Geddes et al. 2014; Yoccoz 1991).

Scientists across disciplines have recently discussed alternatives to *p*‐values. In 2016, The American Statistical Association (ASA) issued a formal statement on *p*‐values, clarifying what they are and what they can measure (see Greenland et al. 2016; Wasserstein and Lazar 2016). Following the Wasserstein and Lazar (2016) publication, the authors published an even bolder editorial that took the next step by recommending the total abandonment of “statistical significance,” including its variants such as “significantly different,” “*p* < 0.05,” and “nonsignificant” (Wasserstein et al. 2019). In response, the ASA created a task force to clarify the use of *p*‐values and provide principles of statistical inference, arguing that *p*‐values remain a valuable tool when applied correctly and in conjunction with other statistical methods; such as including measures of evidence for the hypothesis alongside the *p*‐value as a measure of certainty (Benjamini et al. 2021).

Ecologists and statisticians have responded to criticisms of the misuse and overreliance of the *p*‐value with recommendations to ensure robust statistical reporting, often encouraging multiple modes of inference (Erickson and Rattner 2020; Goodman et al. 2019; Halsey 2019; Krueger and Heck 2019). Published ecological literature is dominated by *p*‐values as a binary metric for significance testing (Muff et al. 2022), but the extent to which non‐frequentist modes of inference have been used over time remains unclear. Moving to a world beyond “*p* < 0.05” requires broader familiarization with alternative modes of inference.

### 1.1 Modes of Statistical Inference

Current literature has suggested multiple modes (or paradigms) of inference, and debated the use of each in different situations (Bandyopadhyay and Forster 2011; Geiser 1993). In a frequentist mode of inference, the probability that the data, or more extreme data, would be observed given the null hypothesis is referred to as the *p*‐value (Greenland et al. 2016). Under this framework, the strength of evidence for the alternate hypothesis is not directly measured (Royall 1997). Likelihood, or Evidentialist, inference asks “what is the likelihood of the hypothesis given the results?” With likelihood inference, the data, or results, are constant, while the hypothesis is the variable. A likelihood‐based mode of inference measures the strength of the evidence for one model relative to another. The model can be either a null (i.e., no difference between groups) or can be the best‐fitting model compared to others (a goodness of fit approach; see McCullagh and Nelder 1989, 24). Bayesian inference, also referred to as priorist or belief‐based inference (Hacking 1965; Schneider 2021), is also based on likelihoods. Evidentialist inference rests on a two‐part inference base (Birnbaum 1962): a sampling model and data taken to have been generated by that model. Frequentist and Bayesian/Priorist inference rest on a three‐part base; for frequentist inference the third component is the law of large numbers, and for Bayesian/Priorist the third component is a prior probability according to Laplace's principle of inverse probability (Stigler 1986). Because Bayesian/Priorist inference in ecology has been more extensively covered by others (e.g., Anderson et al. 2001; Dixon and Ellison 1996; Ellison 2004; Pappalardo et al. 2020), we focus on the use of likelihood ratios in ecology and evolution here.

The term “evidentialist inference” is preferable to the “likelihoodist inference” in that frequentist and Bayesian inference are also founded on likelihood. The more appropriate term “evidentialist” comes from a review (Vieland and Hodge 1998) of Royall's book (Royall 1997) that makes the case for direct likelihood inference. Evidentialist inference is based on the law of likelihood (Hacking 1965), which states that likelihood ratios measure the relative strength of evidence (Royall 1997). Likelihood inference is based on the likelihood function and on the use of maximum likelihood to estimate parameters such as contrasts of means, regression slopes, and odds ratios.

### 1.2 Evidence vs. Certainty

We can draw a distinction between the evidence supporting a particular hypothesis (likelihood) and the certainty of the hypothesis, such as the Type I error measured by a *p*‐value derived from repeated sampling (Edwards 1972; Hacking 1965). The conceptual divide between likelihood and probability goes back to Laplace (1774). Laplace defined two conditional probabilities: the probability of the events given the cause *p* (E | C) and the likelihood, the probability of the cause given the events *p* (C | E). Nearly 250 years after Laplace, Fisher (1922) defined likelihood as the probability of a parameter (*θ*) given the data (*x*), now denoted as ℒ (*θ* | *x*), from which we have probability as the chance of future data, given the parameter *p* (*x* | *θ*). Fisher (1922) extended the use of the likelihood ratio to calculate a test statistic that in turn is used to calculate a *p*‐value—the probability of observing data as extreme or more extreme than the observed data. Because *p*‐values focus on the null hypothesis, this frequentist approach is in practice limited to statements about the null—reject or not reject. Rejecting the null does not entail affirming the alternative, as occurs when *p*‐values are reported as if they were a measure of evidence. Texts on direct likelihood inference began appearing at the end of the twentieth century (Azzalini 1997; Pawitan 2001; Severini 2000). The evidentialist approach uses the likelihood ratio to measure the evidential support of one hypothesis compared to another; there is no probability to be updated nor is there a probability being calculated to test a null hypothesis.

Though evidentialist inference is less common than other modes, one way likelihood is used to directly make statistical decisions is information theoretic (Burnham and Anderson 1998) which uses a penalized likelihood (e.g., Akaike Information Criterion) to rank a set of plausible models. This variety of evidentialist inference (Vieland and Hodge 1998) was developed, in part, in reaction to the long‐established problems with widespread reliance on the use of *p*‐values to evaluate scientific studies (Ioannidis 2005). Ecological and evolutionary research in particular often asks observational questions that may be less suited to frequentist significance testing. In ecology we are often interested in model outputs in addition to individual parameter estimates (Anderson et al. 2000; Gerrodette 2011; Halsey 2019).

Measures of uncertainty (*p*‐values) and measures of evidence (likelihood ratios) do not scale in a one‐to‐one fashion. As sample sizes increase, changes to *p*‐values that would dramatically change interpretation may barely change likelihood ratios (Berger and Sellke 1987; Goodman 1993; Kager and Meester 2026). This is especially relevant to ecological and evolutionary studies, where sample sizes can vary considerably. Discrepancies between measures of evidence and certainty, together with the colloquial equivalence of “likelihood” and “probability” in common usage, impede communication of evidence versus belief. In practical applications of biological and environmental study, misunderstandings of this nature can hinder evidence‐based decision making. This can become acute in any policy arena, recognizing that choice of language can be critical. For example, the Intergovernmental Panel on Climate Change (IPCC) published a “likelihood scale” to clarify language in its Fifth Assessment Report, setting up equivalencies between the terms “likelihood” and “probability” (Kause et al. 2022). This scale ranges from exceptionally unlikely (0%–1% probability) to virtually certain (99%–100% probability). Here, we take the distinction between a measure of evidence (a likelihood ratio; LR) and a measure of certainty (a *p*‐value) as critical to ecologically grounded policy.

### 1.3 Navigating the Gap: Two Examples

We provide two examples in the fields of ecology and evolution to illustrate the differences between evidence versus certainty and the utility of reporting both.

San Román et al. (2023) released wild, farmed, and reciprocal F1 hybrid Atlantic salmon fry at three sites in the Garnish River in Newfoundland, Canada, to evaluate family and cross‐specific patterns of recapture/survival, size, sex ratio, and precocial male maturation over a 28 month period. These authors used goodness of fit tests of the number of individuals recaptured per family relative to the expected number of recaptures. In a factorial design, cross type, site, year, and all the possible interaction terms were taken as fixed factors in the model. These authors assessed the certainty of the data (the *p*‐value) alongside the relative magnitude of evidence for an alternative hypothesis, with LR > 10 indicating strong evidence, LR > 32 substantial evidence, and LR > 100 decisive evidence (Jeffreys 1961; Royall 1997). As an example, they reported that in 2018 and 2019 there was evidence for an interaction between site and cross type (2018: *p* = 0.079, LR = 289.3; 2019: *p* < 0.001, LR = 5.79 × 10^5^), while in 2020 the evidence was strong based only on the likelihood ratio score (*p* = 0.38, LR = 24.82; see Supporting Information in San Román et al. 2023). Note that the 2020 assessment showed strong evidence for an interactive effect that would have been dismissed if only the *p*‐value were reported. What does it mean that the interaction is likely, without a significant *p*‐value? In this case, the hypothesis that there is an interaction between site and cross type is 25 times more likely than the alternate hypothesis being compared—that there is no interaction. At the same time, there is considerable uncertainty (38%) that these results could be observed under the null hypothesis and that there is no interactive effect. The likelihood of a hypothesis is not synonymous with the certainty that the data would not be observed under the null. Reporting both metrics provides more information and context for the results.

The second example is from a lab experiment in the context of climate change (Shellem et al. 2025). The analysis of deviance table (Table 1, reproduced from table 2 in Shellem et al.) shows the results of the generalized linear model of sea star righting time at two temperatures and two salinities over 4 days. The *F*‐ratio for the salinity × temperature term in Table 1 is 4.11, for which the *p*‐value is 0.05 on 1 and 152 degrees of freedom. The likelihood ratio calculated from the deviance, reported in the table, is 1.8 (when back‐calculated from the *F*‐ratio, LR = 9.51; see Appendix S1 for LR formulas), which would be considered inadequate evidence, since LR > 10 is a suggested threshold for “strong” evidence (Jeffreys 1961; Royall 1997). The likelihood ratios and *p*‐values both make it evident that salinity alone was important. There was limited evidence for a temperature and salinity interactive effect despite the small *p*‐value, which is very near significance under *α* < 0.05. In this case, the LR provides further information to determine the importance of an interaction effect. This makes the decision to ignore the interaction effect better supported despite the *p*‐value being very near the alpha threshold.

**TABLE 1:** Statistical results from Shellem et al. (2025).

	Δdf	Δdeviance	df residual	Residual deviance	*F*‐ratio	*p* from *F*	LR from deviance
NULL			158	56.51			
Temp.	1	0.075	157	56.43	0.26	0.61	1.04
Salinity	1	15.13	156	41.31	51.56	**< 0.001**	1925
Day	3	0.33	153	40.98	0.37	0.78	1.18
Temp.:Salinity	1	1.2	152	39.78	4.11	0.05	1.83
Temp.:Day	3	1	149	38.77	1.14	0.35	1.65
Salinity:Day	3	0.94	146	37.84	1.07	0.38	1.6
Temp.:Salinity:Day	3	0.65	143	37.18	0.74	0.54	1.39

*Note:* Significant *p*‐values are bolded.

These two examples differ in the way that evidence and uncertainty were reported. The salmon study used a stated set of criteria for strength of evidence to draw conclusions. Uncertainty was reported for the use of the reader, consistent with the recommendations of Benjamini et al. (2021). The sea star study drew conclusions from uncertainty in tandem with the LR. Stated criteria were not used, there being no evidence for any effect other than salinity. In both examples, the use of both evidence and uncertainty permitted a more informed conclusion than one based only on a pre‐set level of uncertainty alone. Moreover, in a pedagogical setting, for science students and for this journal, the evidentialist form (LR ranging upward with stronger evidence) is more intuitive and readily grasped than the convention of LR ranging downward from 1 for the better fitting model.

These examples illustrate how different conclusions could be drawn and different management decisions made based on the mode of inference used. Here, we are interested in the application of likelihood ratios in addition to *p*‐values (as recommended by Benjamini et al. 2021). We quantified whether, and to what degree, frequentist (*p*‐values) and evidentialist (likelihood ratio) conclusions differ in the published ecological and evolutionary literature at three time periods. We investigated the source of discrepancies between the two modes of inference, particularly in terms of the test statistic used. We then further explored whether there have been changes in the use of *p*‐values and likelihood ratios over time. To put trends in context, we extended the comparison to the use of other likelihood‐based modes of inference, Bayesian and the use of generalized likelihood (AIC). Finally, we analyzed whether there has been a decline in the reporting of *p*‐value thresholds over time, despite repeated recommendations to report exact *p*‐values (Goodman 1993; Wasserstein and Lazar 2016), through the late 20th century and into the 21st.

## 2 Methods

To investigate the use of different modes of statistical inference and their consequences, we extracted data from five scientific journals, selected based on the fields the authors primarily worked in (*Animal Behavior*, *Biological Conservation*, *Canadian Journal of Fisheries and Aquatic Sciences*, *Landscape Ecology*, and *Marine Ecology Progress Series*). To quantify temporal patterns, we arbitrarily collected data from the second issue of each journal at a decadal interval (i.e., 1999, 2009, and 2019; 130 publications). We investigated: (a) which mode of inference was used (Frequentist, Evidentialist, and Bayesian), (b) whether the article provided a rationale for the given choice, (c) if sufficient information was provided to calculate a likelihood ratio, and (d) if the conclusions drawn from a calculated likelihood ratio using the data were the same as the conclusions stated in the article derived from a *p*‐value. Further, we explored the sources of discrepancy between these *p*‐values and LRs.

Likelihood ratios were obtained by back calculation from measures of fit: *R* ^2^, *F*‐ratios and *t*‐statistics, and chi‐square/*G* statistics (Appendix S1). If there was insufficient information from these test statistics, they were obtained from sums of squares in normal error ANOVA tables and from changes in deviances in ANODEV tables when available (Appendix S1). The negative log of *p*‐values were then plotted against the log of likelihood ratios (Figure 1). If *p*‐values were reported as thresholds (e.g., *p* < 0.01), a likelihood ratio was still calculated, but the datapoint was excluded from Figure 1. The number of threshold values compared to exact *p*‐values was recorded (Figure 2).

**FIGURE 1:**
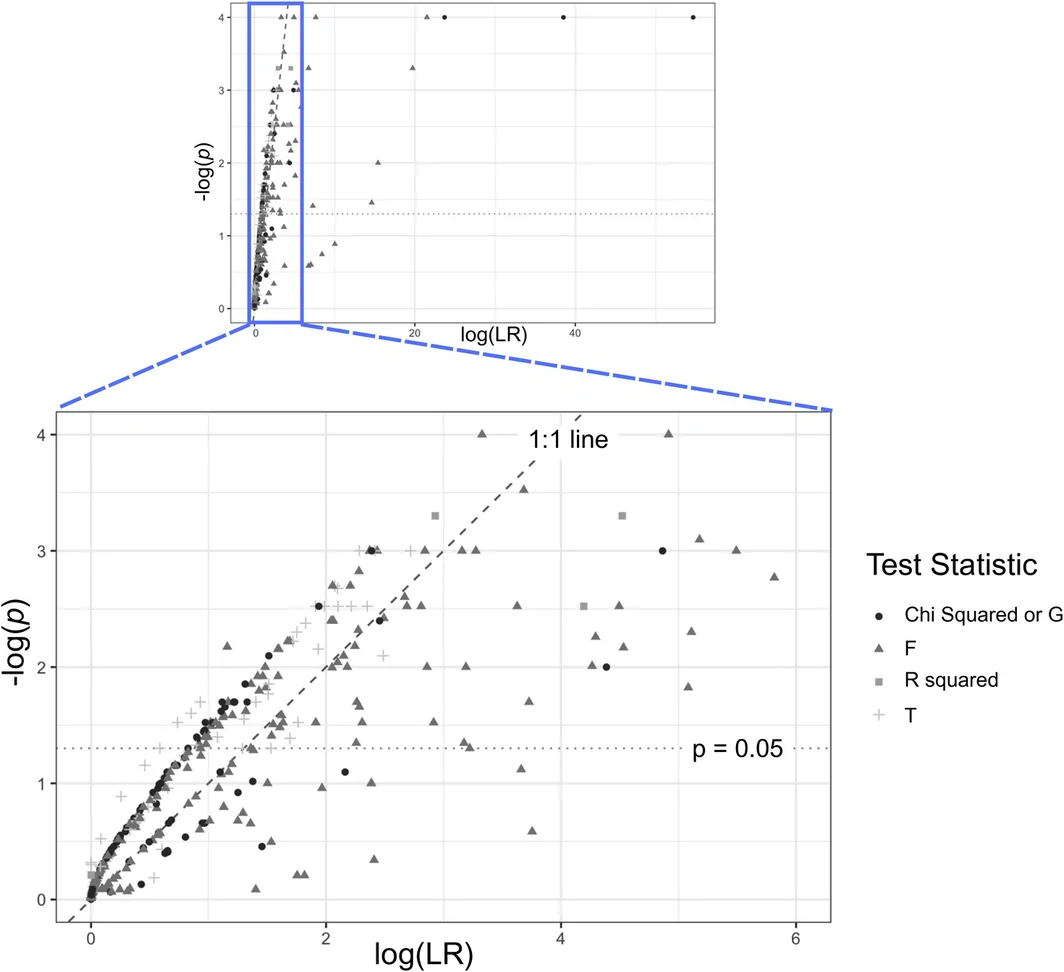
Calculated likelihood ratio compared to the reported *p*‐value from the literature review on a base‐10 log scale (above), zoomed to show detail (below). Shade and shapes represent the test used. The dashed line represents *x* = *y* (1:1 line). The dotted line represents *p* = 0.05; all points above this line on the *y*‐axis have a *p*‐value less than 0.05. Points in the upper left represent high certainty but low evidence (low *p*‐value, low LR), while points in the lower right represent high evidence but low certainty (high *p*‐value, high LR).

**FIGURE 2:**
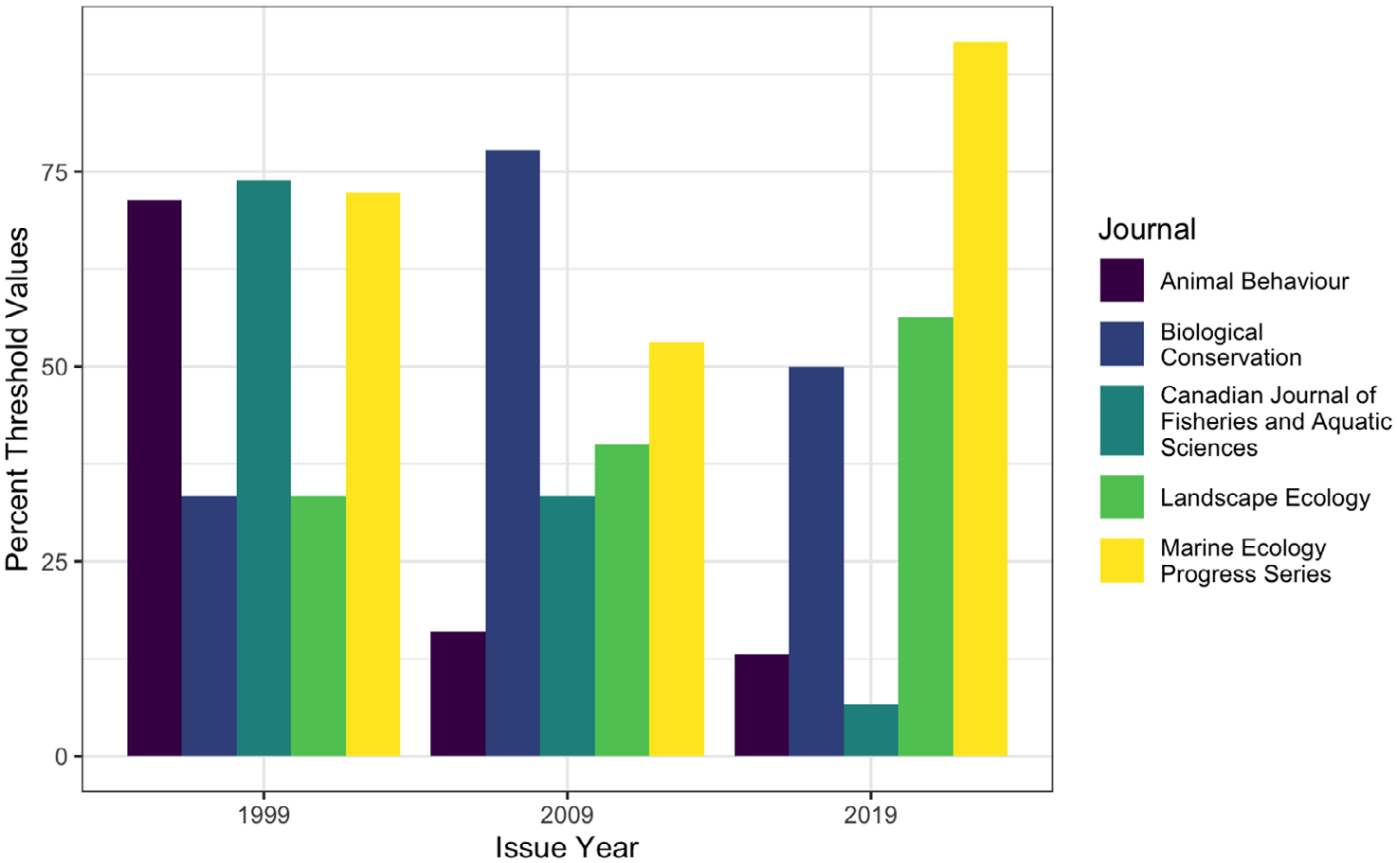
Percent of *p*‐value threshold values at each time point by journal.

## 3 Results and Discussion

In total 1084 test statistics were compiled from 130 publications. Of those, we had sufficient information to calculate LRs from 855 test statistics, and 79 publications included rationale for the mode of inference used for at least one test statistic. We expected that likelihood ratios would not scale in a linear fashion with *p*‐values based on previous research (Berger and Sellke 1987; Goodman 1993; Kager and Meester 2026). We found this to be true in the ecological and evolutionary literature, particularly for *F*‐statistics (Figure 1).

### 3.1 Divergence Between Likelihood Ratios and *p*‐Values

Within the published literature, conclusions drawn from reported *p*‐values often differed from those based on the likelihood ratios used to obtain *p*‐values (Figure 1). Generally, the negative log of *p*‐values and log of likelihood ratios scaled in a monotonic fashion, falling near a 1:1 line. However, there were notable exceptions. Some test statistics had high likelihood ratios but high *p*‐values. This trend was particularly visible for *F*‐statistics (Figure 1 triangle symbol ▲). Many of these points diverged strongly from a one‐to‐one relationship. In contrast, chi‐square and *t*‐tests can be seen to place higher certainty on little evidence; the points that fall above (to the left of) a one‐to‐one line are almost entirely *t*‐tests (Figure 1 plus‐sign symbol +).

These results show that tests with high degrees of freedom in the numerator (and a large ratio of df numerator to df denominator) for the *F*‐statistic were more likely to have high evidence where certainty was low (Figure S1). In extreme cases, we found tests that had strong evidence (LR > 100) with *p*‐values that would be considered insignificant (*p* > 0.05); all but one of these cases were derived from *F*‐statistics (*n* = 10, Figure 1). Conversely, we found cases with *p*‐values that would be considered significant (*p* < 0.05) yet had comparatively little evidence (e.g., LR < 10, Jeffreys 1961; Royall 1997; *n* = 15). Our results document the prevalence of inconsistent scaling of likelihood ratios relative to *p*‐values demonstrated by Kager and Meester (2026) across a subset of journal issues encompassing ecological and evolutionary studies. This discrepancy shows that evidence and certainty measures are not identical, and one cannot simply be substituted for the other. In other words, our results show that a particular hypothesis can have substantial evidence from the data compared to another hypothesis (a high LR), while also having a high probability that the same data would be observed under a null hypothesis (high *p*‐value). This situation might occur if there is a large effect on a small sample size, for example, or when a large number of random factors with little explanatory value reduce the residual degrees of freedom, lowering the significance of the effect (Figure S1). A high LR and high *p*‐value were most prominent among *F*‐statistics in our sample. Conversely, a hypothesis could have a very low probability of the data being observed under a null hypothesis (low *p*‐value) but low relative evidence of that hypothesis. This was most commonly found for *t*‐tests in our sample. A very low likelihood but significant *p*‐value can commonly occur when there is a small effect size but a very large sample size. This distinction implies that the evidence for one hypothesis over another and the probability that the data would be observed under the null hypothesis measure different, though related, inferential metrics. In many of these cases, other conclusions could have been drawn if a different mode of inference was used. This divergence could have profound implications for conservation and ecological management.

### 3.2 Changes in Modes of Inference Over Time

In the review of 130 published articles in five journals over three decades, we found 806 examples of frequentist inference and 10 examples of Bayesian inference (Table 2). A preliminary screening produced seven publications identified as evidentialist. Of these seven articles, six discussed likelihood by implementing the use of AIC for model selection, while only one publication reported a LR as a measure of evidence. The value of the measure of evidence was limited in that it was not reported along with a measure of uncertainty. Additionally, frequentist inference dominated the publications examined throughout the past three decades (Table 2), consistent with the results found in Muff et al. (2022).

**TABLE 2:** Count of published test statistics using each mode of inference by each year investigated.

Year	Bayesian	Frequentist	Evidentialist
1999	6	313	0
2009	2	314	3
2019	2	179	4

We also found downward trends in the use of *p*‐value thresholds (i.e., “*p* < 0.05”) over time in two of the five journals surveyed (Figure 2). Despite recommendations that exact *p*‐values, not threshold values, be reported (Goodman 1993), three journals showed no consistent decrease in reporting of thresholds from 1999 to 2019.

Here our results represent a preliminary investigation, using a small subset of journals across three time periods. We did not investigate differences in study design, model type (fixed vs. random factors), or differences in the extent of the discrepancy by subfield. Future research in this area should conduct a more detailed analysis with a larger sample size to better understand the extent of evidence and certainty divergence to better understand the implications for publication bias and the replication crisis.

The discrepancy found between likelihood ratios and *p*‐values has implications for scientific replicability, particularly related to the “file drawer effect” (Rosenthal 1979). Publication bias is a significant problem in ecology and evolution, with published studies showing low statistical power, low sample size, and meta‐analyses failing to account for this (Yang et al. 2023, 2024). Multiple data points in our study had *p*‐values below 0.05 but low likelihood (LR < 10; Figure 1). Additionally, we found ten points with very strong evidence (LR > 100) but an insignificant *p*‐value (Figure 1). Our results lead us to hypothesize that these types of statistics may be systematically less likely to be published due to the lack of statistical significance, despite high likelihood. Future research should investigate these impacts on replicability and publication bias. Reporting likelihood ratios in addition to *p*‐values would provide more transparency on statistical significance and allow the reader to better understand the study's findings separate from the authors' interpretations and conclusions.

### 3.3 Recommendations and Conclusion

Our results quantify the discrepancy between frequentist *p*‐values and evidentialist likelihood ratios. Additionally, we show that the discrepancy established in theoretical work (Berger and Sellke 1987; Goodman 1993; Kager and Meester 2026) is frequent and can be of substantial magnitude in the published literature. Based on these results, we bring forward the following recommendations:

1. Adding to previous research, our results reinforce the relevance of separating probability and evidence. Therefore, we recommend reporting both a measure of evidence (likelihood ratio) and a measure of uncertainty (*p*‐value, confidence limit, etc.). This is especially important when handling *F* statistics with a high ratio of degrees of freedom in the numerator compared to the denominator, where results with insignificant *p*‐values may be underreported despite substantial evidence. Our recommendation that a measure of evidence be reported along with a measure of uncertainty (the *p*‐value) is similar to the “rules of acceptance” (Mayo 1996, 404), which take into account effect size when a null hypothesis cannot be rejected.
2. Extending the recommendations from Yoccoz (1991), we encourage the reporting of exact *p*‐values, not thresholds. Small differences in *p*‐values can reflect large differences in likelihood (Kager and Meester 2026). This also reaffirms that uncertainty on an estimate should be reported exactly, instead of a binary “significant or not significant” decision. We encourage journal editors to mandate this when possible, since the prevalence of threshold reporting varies for each journal. In addition, we recommend that authors always provide sufficient information to reproduce statistics including effect size, number of observations or degrees of freedom, test statistic, measure of evidence (likelihood ratio) or measure of fit, and measure of uncertainty (*p*‐value, confidence interval, etc.). This has been identified as a significant problem in science in general, as well as throughout the field of ecology and evolution (Anderson et al. 2001; Parker et al. 2016).
3. We recommend that authors state their mode of inference and the reasoning for their choice to provide clarity of language surrounding results. In certain situations, when Type I error must be controlled (e.g., environmental impacts such as contaminant releases, conservation of species at risk) using established risk levels with a frequentist mode of inference and statistical cutoffs may be the most appropriate. In many cases, notably observational studies, likelihood inference may be the most fitting and should be considered. This is especially relevant when using high‐powered *F* statistics.

For many problems in ecology, a frequentist framework alone is insufficient. It is difficult to form meaningful null hypotheses to be rejected for a myriad of questions, ranging from “how many animals are in this area?” to “which genes are involved in adaptation to the environment?”, and inference would be better suited to methods that focus on the likelihood of each hypothesis, model, or estimate, alongside a measure of uncertainty (see Chamberlin 1965). In many situations, *p*‐values provide utility, particularly when the cost of underestimating Type I error is high. In ecological fields such as conservation biology, the social and environmental costs of inaction are often severe—including climate change, anthropologic development, and extinction. Reporting both evidence and certainty can improve understanding, clarify results, and avoid the pitfalls of reporting only a measure of uncertainty. Ultimately, by combining *p*‐values as a statistical filter to ensure a signal is not random noise with likelihood ratios to weigh the relative strength among competing hypotheses, you build a framework that controls for false positives while identifying the most probable hypothesis based on observed evidence.

## Author Contributions

**Hallie E. Arno:** conceptualization (equal), data curation (equal), formal analysis (equal), investigation (equal), software (lead), visualization (equal), writing – original draft (lead), writing – review and editing (equal). **Robert J. Blackmore:** conceptualization (equal), data curation (equal), investigation (equal), methodology (equal), writing – original draft (supporting), writing – review and editing (equal). **Samantha E. Crowley:** conceptualization (equal), data curation (equal), investigation (equal), methodology (equal), writing – review and editing (equal). **S. M. R. Evon:** data curation (equal), validation (lead), writing – review and editing (equal). **Ian A. Fleming:** conceptualization (equal), supervision (equal), writing – review and editing (equal). **C. Tyler Fountain:** conceptualization (equal), data curation (equal), investigation (equal), methodology (equal), writing – original draft (supporting), writing – review and editing (equal). **Stephan Hamisch:** conceptualization (equal), data curation (equal), investigation (equal), methodology (equal), writing – original draft (supporting), writing – review and editing (equal). **Emily M. Monk:** conceptualization (equal), data curation (equal), investigation (equal), methodology (equal), writing – original draft (supporting), writing – review and editing (equal). **Alec L. Robitaille:** conceptualization (equal), data curation (equal), investigation (equal), software (supporting), writing – review and editing (equal). **David C. Schneider:** conceptualization (lead), funding acquisition (equal), methodology (lead), supervision (equal), supervision (equal), writing – original draft (supporting), writing – original draft (supporting), writing – review and editing (equal), writing – review and editing (equal).

## Funding

This work was supported by the German Academic Scholarship Foundation (N/A), Canadian Network for Research and Innovation in Machining Technology, Natural Sciences and Engineering Research Council of Canada, Science Undergraduate Research Award (SURA), President's Award for Graduate Teaching at Memorial (N/A), and Foundation for the Conservation of Atlantic Salmon (SAC‐2023‐02).

## Conflicts of Interest

The authors declare no conflicts of interest.

## Supporting information




**Appendix S1:** Likelihood ratios measure relative evidence.

**Figure S1:** Calculated likelihood ratio compared to the reported *p*‐value from the literature review on a base‐10 log scale for *F*‐statistics only. Colors represent df numerator to df denominator ratio, and size represents the value of the degrees of freedom in the numerator. The dashed line represents *x* = *y* (1:1 line). The dotted line represents *p* = 0.05; all points above this line on the *y*‐axis have a *p*‐value < 0.05. Points in the upper left represent high certainty but low evidence (low *p*‐value, low LR), while points in the lower right represent high evidence but low certainty (high *p*‐value, high LR).

**Data S1:** ece374419‐sup‐0003‐DataS1.zip.

## Data Availability

All the required data are uploaded as Supporting Information. Additionally, the data and code are stored in a Github repository here: https://github.com/hallie‐arno/ModesOfInference. The repository is also available on zenodo: https://doi.org/10.5281/zenodo.23344699.
