# Supplementary material for: p‐Values Are Not Evidence: Navigating the Gap Across Modes of Inference
Source: Ecol Evol. 2026 Oct 25;16(10):e74419. doi: 10.1002/ece3.74419 (PMC13710907; doi:10.1002/ece3.74419)
Supplement: Supplementary file 1 [file ECE3-16-e74419-s003.docx]

Appendix A. Likelihood ratios measure relative evidence.

They can be calculated from goodness of fit statistics and from null hypothesis test statistics.

A. Goodness of fit tests. *LR* ranges upward from 1 (no evidence) to stronger evidence

$LR= e^{LRSt/2}$	*LRSt* = $\chi_{o}^{2}$ , $G$ , $G^{2}$, $D^{*}$

$\chi_{o}^{2}= -2\log\lambda_{c}$	Wilks (1935) Likelihood Ratio Statistic, based on

Wilks Likelihood Criterion	0 < $\lambda_{c}<1$

Applications: Fit to theory. Heterogeneity in two way table.

$\chi_{o}^{2}$ is distributed as $\chi^{2}$ with $k$ degrees of freedom $\chi_{o}^{2} \sim\chi^{2}(k)$ Wilks 1938.

$G=2\sum_{1}^{k} f ln\left( \frac{f}{\hat{f}} \right)$	Sokal and Rohlf 1969. Fit to theory, 2-way heterogeneity.

*f* = frequency in *k* classes. $\hat{f}=$ fitted value.

$G^{2}= -2\sum x_{observed}ln\left( \frac{x_{expected}}{f_{observed}} \right)$	Bishop et al. p58 multiway tables.

$G^{2}= 2\sum x_{observed}ln\left( \frac{x_{observedd}}{f_{expecteded}} \right)$	$x=$ count. Poisson error

$D^{*}$ the change in deviance (improvement in fit) in the ANODEV table

$D^{*}=-2ln(L_{full}/L_{reduced} )$	McCullagh and Nelder 1989 p 24

$L_{full}$ is the likelihood of the full (saturated) model

$L_{reduced}$ is the likelihood of the reduced model

$D^{*}\sim\chi^{2}(k)$	McCullagh and Nelder 1989 p 471

$LR=(L_{m}/L_{full} )$	Hardin and Hilbe 2012 p 48

$L_{m}$ i is fitted model, $L_{f}$ i is full (saturated) model

$LR= e^{-G/2}$	For reporting fitted relative to saturated

B. Null hypothesis tests. *LR* ranges downward from 1 to better evidence.

Take the inverse to compare alternative hypothesis to the null hypothesis.

From the explained variance *R^2^*	Hardin and Hilbe 2012 p60

Normal error only. All parameters relative to intercept.

$LR={(1-R^{2})}^{n/2}$	For reporting null relative to the alternative

$LR={(1-R^{2})}^{-n/2}$	For reporting alternative relative to the null

From the *F* statistic.	Azzalini 1996 p 131.

$LR= \left( 1+F\left( \frac{{df}_{numerator}}{{df}_{denominator}} \right) \right)^{n/2}$	For reporting alternative relative to the null

From the *t* statistic	From *F*-statistic with numerator *df* = 1.

$LR= \left( 1+{t^{2}}/\left( n-2 \right) \right)^{n/2}$	For reporting alternative relative to the null

For fixed terms displayed vertically in an ANOVA table.

$LR= \left( \frac{{SS}_{term}+{SS}_{residual}}{{SS}_{residual}} \right)^{n/2}$	For reporting alternative relative to the null

For generalized linear models.

$W^{2}=2 \mathcal{l}\left( \hat{\theta} \right)-2\mathcal{l}\left( \theta_{o} \right)$ = $\Delta Deviance$	McCullagh and Nelder 1989 p 476

$W^{2}$ = Log likelihood statistic for testing $H_{o}: \theta=\theta_{o}$

$\mathcal{l()}$ is the likelihood function.

$LR= e^{W^{2}/2}$	For reporting alternative relative to the null

References

Azzalini, A. (1996). *Statistical Inference. Based on the Likelihood*. Boca Raton: Chapman and Hall.

Bishop, Y.Y., S.F. Feinberg, & P.W. Holland. (1975). *Discrete Multivariate Analysis*. Cambridge MA: MIT Press.

Hardin J.W, & J.W. Hilbe. 2012. *Generalized Linear Models and Extensions*. College Station, Texas: Stata Press.

McCullagh, P. & J,A, Nelder. (1989). *Generalized Linear Models*. Boca Raton: Chapman and Hall.

Sokal, R.R. & F.J. Rohlf. (1969). *Biometry.* San Francisco: Freeman.

Wilks, S. S. (1935). The likelihood test of independence in contingency tables. *Annals of Mathematical Statistics* 6(4), 190–196. DOI: 10.1214/aoms/117773256

Wilks, S.S. (1938). The large-sample distribution of the likelihood ratio for testing composite hypotheses. *Annals of Mathematical Statistics* 9(1): 60-62. DOI: 10.1214/aoms/1177732360
